# Classifying Heart-Sound Signals Based on CNN Trained on MelSpectrum and Log-MelSpectrum Features

**DOI:** 10.3390/bioengineering10060645

**Published:** 2023-05-25

**Authors:** Wei Chen, Zixuan Zhou, Junze Bao, Chengniu Wang, Hanqing Chen, Chen Xu, Gangcai Xie, Hongmin Shen, Huiqun Wu

**Affiliations:** 1 Medical School, Nantong University, Nantong 226001, China; chenwei0303@ntu.edu.cn (W.C.);; 2 School of Information Science and Technology, Nantong University, Nantong 226019, China

**Keywords:** heart-sound classification, MelSpectrum, CNN, STFT

## Abstract

The intelligent classification of heart-sound signals can assist clinicians in the rapid diagnosis of cardiovascular diseases. Mel-frequency cepstral coefficients (MelSpectrums) and log Mel-frequency cepstral coefficients (Log-MelSpectrums) based on a short-time Fourier transform (STFT) can represent the temporal and spectral structures of original heart-sound signals. Recently, various systems based on convolutional neural networks (CNNs) trained on the MelSpectrum and Log-MelSpectrum of segmental heart-sound frames that outperform systems using handcrafted features have been presented and classified heart-sound signals accurately. However, there is no a priori evidence of the best input representation for classifying heart sounds when using CNN models. Therefore, in this study, the MelSpectrum and Log-MelSpectrum features of heart-sound signals combined with a mathematical model of cardiac-sound acquisition were analysed theoretically. Both the experimental results and theoretical analysis demonstrated that the Log-MelSpectrum features can reduce the classification difference between domains and improve the performance of CNNs for heart-sound classification.

## 1. Introduction

Cardiovascular diseases (CVDs) are one of the major threats to human health [1]. Generally, doctors use a stethoscope (placed over what are called cardiac auscultation points) to determine the presence of certain CVDs. With the development of modern medical equipment technology, echocardiography and computed tomography (CT) are more accurate and comprehensive in diagnosing heart diseases than a stethoscope, but they are also more time-consuming and expensive. Consequently, they are not suitable for large-scale preliminary examination, especially in rural areas and grass-roots communities with insufficient medical resources.

Heart sounds, physiological signals generated by myocardial contractions, have important clinical value in the prevention and diagnosis of CVDs, because they can reflect information about cardiovascular hemodynamic changes [2]. Usually, patients who have damaged the structure of the heart valve or exhibit abnormal heart function do not show clinical symptoms initially. Changes in the structure of the heart valves directly lead to narrowing of the blood vessels, increased blood flow, or abnormal channels between the arteries and veins, which, in turn, cause blood turbulence and produce murmurs. Consequently, automatic classification and recognition of heart-sound signals is of great importance for the prevention and diagnosis of CVDs. Up to now, an increasing number of artificial intelligence (AI) techniques have been used to automatically diagnose CVDs with the help of heart sounds [3,4].

In particular, feature extraction is very important in the classification process of heart-sound signals [5]. When classifying heart-sound signals, it is common to transform the raw one-dimensional heart sound signals into two-dimensional features using a time-frequency analysis method, and then use these two-dimensional features to train the convolutional neural networks (CNNs). Some time-frequency analysis methods have been applied to examine heart-sound signals, such as STFT and continuous wavelet transformation (CWT) [6,7,8,9,10,11]. Specifically, STFT has been the most widely used method for research on non-stationary signals. The basic idea of STFT is to use a time-sliding analysis window to truncate non-stationary signals, decompose them into a series of approximately stationary signals, and then use the Fourier transform theory to analyse the spectrum of each short-time stationary signal. In addition, it is easy to implement on hardware platforms, has practical application value in embedded systems, and has real-time requirements. Therefore, research results can be easily applied to smart wearable biosensors [12,13,14].

Usually, original heart-sound signals are generally transformed into two-dimensional feature maps that offer a rich representation of the temporal and spectral structures of the original heart-sound signals. These feature maps are then used to train deep learning neural networks. The most commonly used features are Mel-frequency cepstral coefficients (MelSpectrums) [15,16,17] and log Mel-frequency cepstral coefficients (Log-MelSpectrums) [18,19,20]. These features are based on STFT. Systems based on CNNs trained on MelSpectrums and Log-MelSpectrums of segmental heart-sound signals are superior to other systems using hand-crafted features [21,22,23,24,25,26].

For instance, Deng et al. [22] introduced a novel feature extraction method based on MelSpectrums to represent the dynamics of heart sounds, which were fed to a fused model combining a CNN and recurrent neural network (RNN) for classifying heart sounds. An accuracy of 98% was obtained when classifying normal and abnormal heart sounds. In addition, Nilannon et al. [25] combined MelSpectrums and spectrogram feature maps from fixed S5 heart-sound signals to train a CNN model and this method obtained an accuracy of 81.1%. Abdollahpur et al. [27] extracted 90 features in the time, frequency, perceptual, and Mel-frequency domains from segmented cycles of heart-sound signals. Three feed-forward neural networks combined with a voting system were used to perform the heart-sound classification task. Cheng et al. [28] presented a lightweight laconic heart sound neural network model that has low hardware requirements and can be applied to mobile terminals. This model was implemented using a two-dimensional spectrogram of heart sounds with a 5 s time period. Hence, this study has positive significance for recognising when to train deep learning neural networks. For instance, Rubin et al. [29] used Spring heart sounds in real life.

Conversely, some recent studies used Log-MelSpectrum featurer’s segmentation algorithm [30] to fix heart-sound signals with 3 s segments and convert them into two-dimensional MelSpectrum feature maps. Maknickas and Maknickas [19] proposed a CNN-based model and trained it using Log-MelSpectrum features. The trained model produced an average classification accuracy of 86.02% for recognising normal and abnormal heart-sound frames. Nguyen et al. [31] suggested a long-term memory and CNN model trained using Log-MelSpectrum. The proposed model can classify five different heart sounds. In addition, Li et al. [32] improved Log-MelSpectrum feature maps using dynamic and static MelSpectrum features, and used them as input features for deep residual learning. This method obtained an accuracy of 94.43% for the fusion datasets of three different platforms. 

In general, these different time-frequency features based on STFT were implemented for heart-sound classification and have made a substantial contribution. However, there is no a priori evidence of the best input representation for classifying heart sounds when using deep learning models. To solve this problem, MelSpectrum and Log-MelSpectrum features of heart-sound signals combined with the mathematical model of heart cardiac-sound acquisition were analysed theoretically in this study. In addition, these two features were input to a general CNN model to evaluate further the features that are more suitable for classifying heart-sound signals.

To our knowledge, this is the first study that has analysed theoretically the MelSpectrum and Log-MelSpectrum features of heart-sound signals to determine which one is more suitable for classifying heart-sound signals when using CNNs. In addition, our study provides the following major contributions to existing literature. First, by analysing the mathematical model of cardiac-sound acquisition, we conclude that the MelSpectrum and Log-MelSpectrum feature maps as input feature vectors of the CNN are efficient for additive and multiplicative noise suppression, respectively. Second, we evaluated our method based on published datasets from the PhysioNet/CinC Classifying Heart Sounds Challenge [33]. The MelSpectrum and Log-MelSpectrum features were input to a CNN-based model to classify heart-sound signals, and the experimental results showed that MelSpectrum and Log-MelSpectrum as input features of the CNN can be used as effective methods for classifying heart sounds. Furthermore, compared with MelSpectrum features, Log-MelSpectrum features are more suitable for processing heart-sound datasets that have domain differences and for improving the performance of CNN for heart-sound classification.

## 2. MelSpectrum and Log-MelSpectrum Features

### 2.1. Extraction of MelSpectrum and Log-MelSpectrum Features

**Figure 1:**
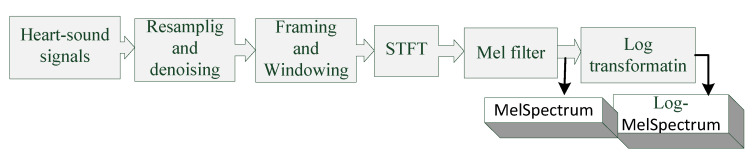
Time and frequency feature extraction process.

Mel filter, a useful tool for processing speech signals, has been widely applied in automatic speech recognition (ASR). It can reflect the non-linear relationship between human hearing and the sound heard. Recently, various studies have used Mel filters to extract valuable features from heart-sound signals, and the MelSpectrum and Log-MelSpectrum discussed herein are based on Mel filters. The parameters of MelSpectrum and Log-MelSpectrum in our study are shown in Table 1 and the feature extraction process is shown in Figure 1. The detailed process of feature extraction is described as follows:

1. The heart-sound signals are resampled from 25 Hz to 950 Hz using a Butterworth filter with a sampling frequency of 2000 Hz. The signals are then passed through a Savitzky–Golay filter to improve the smoothness of the time-frequency feature graph and reduce noise interference.
2. The filtered signals are framed and windowed using a Hanning window function to fix the signals into a selected frame length.
3. Frames are transformed into the periodogram estimate of the power spectrum using STFT.
4. Each periodogram estimate is mapped onto the Mel-scale using Mel filters, which consist of several triangular filters. The output of the Mel filter is called the MelSpectrum.
5. Logarithmic transformation is applied to the MelSpectrum features to obtain the Log-MelSpectrum.

**Figure 2:**
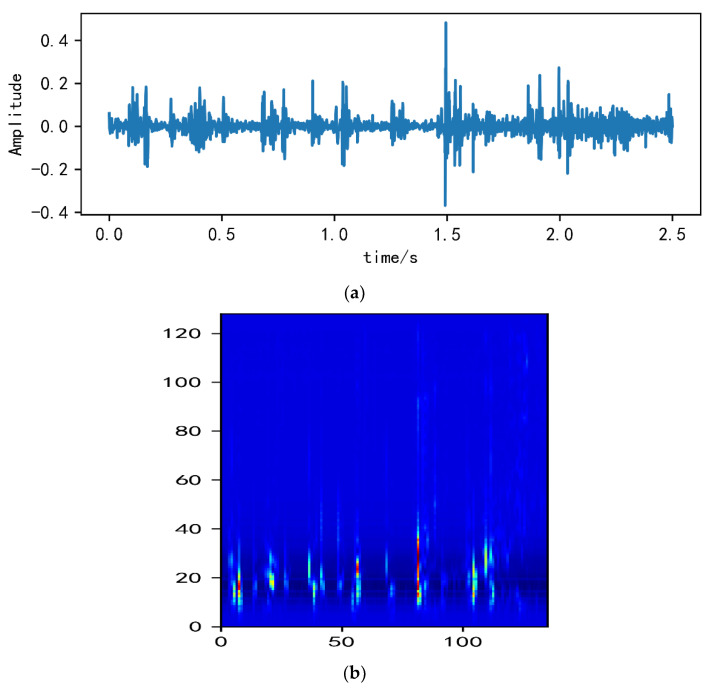

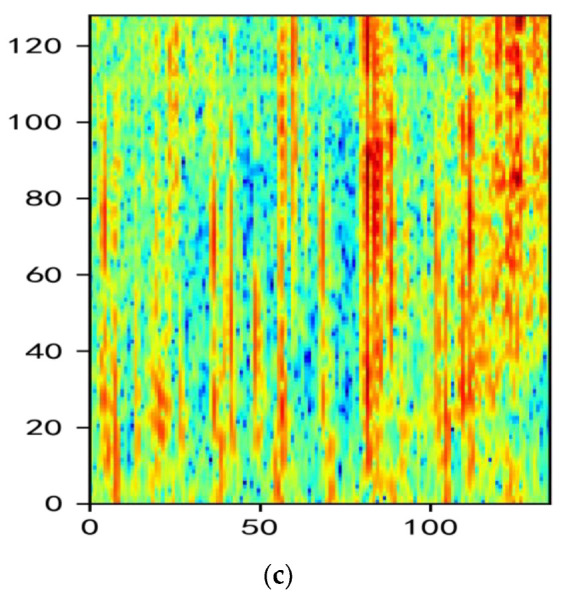
Normal heart-sound signal example: (**a**) Heart-sound signal (.wav file); (**b**) MelSpectrum feature map; (**c**) Log-MelSpectrum feature map.

Examples of MelSpectrum and Log-MelSpectrum feature maps from normal heart-sound fragments are shown in Figure 2. 

### 2.2. Analysis of MelSpectrum and Log-MelSpectrum

**Figure 3:**
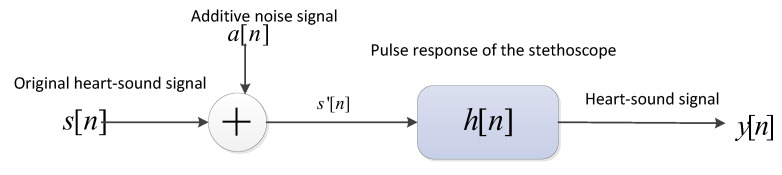
Cardiac-sound collection model.

Heart-sound signals are easily disturbed by additive and multiplicative noise during the acquisition process. Figure 3 shows the mathematical model of cardiac-sound acquisition.

In Equation (1) below, *s*(*n*) is the original heart-sound signal, *a*(*n*) *is* the additive noise signal, including background sounds, breath sounds, lung sounds, and other noises caused by friction between the equipment and the skin, and *h*(*n*) is the pulse response of the stethoscope. The actual collected heart-sound signal *y*(*n*) is given by:

(1) $$y(n)=(s(n)+a(n))*h(n)=\sum_{m=-\infty }^{\infty }\left(s(m)+a(n-m))*h(m\right),$$

where * denotes the convolution operation. The STFT of Equation (1) can be expressed as:

(2) $$Y\left[l,k\right]=\left(S\left[l,k\right]+A\left[l,k\right]\right)H\left[l,k\right]l=1,2,\ldots ,L;k=1,2\ldots K\;.$$

Here, *H*[*l*,*k*] *is* the representation of the impulse response of the stethoscope in the frequency domain; *Y*[*l*,*k*], *S*[*l*,*k*], and *A*[*l*,*k*] are the STFT forms of *y*(*n*), *s*(*n*), and *a*(*n*), respectively; and *l* and *k* are the frame in the time domain and the band in the frequency domain of the heart sound signal, respectively.

Taking the square of Equation (2), we obtain:

(3) $${\left|Y[l,k]\right|}^{2}=\left({\left|S[l,k]\right|}^{2}+{\left|A[l,k]\right|}^{2}\right){\left|H[l,k]\right|}^{2}+2\left|S[l,k]\right|\left|A[l,k]\right|\cos\theta {\left|H[l,k]\right|}^{2}$$

where *θ* is the phase angle between the heart sound and the noise signals. Because *s*(*n*) and *a*(*n*) are independent, the above equation can be expressed approximately as:

(4) $${\left|Y[l,k]\right|}^{2}=\left({\left|{\left|A[l,k]\right|}^{2}+S[l,k]\right|}^{2}\right){\left|H[l,k]\right|}^{2}.$$

The power spectrum estimation results of each frame were filtered by Mel filter banks composed of M triangular filters and a weighted sum with each filter. After the Mel filter process, we obtain the output energy of the filter banks, namely, MelSpectrum that can be expressed as *Mels* variable in the formula:

(5) $$\begin{array}{c} Mels={\left|Y[l,m]\right|}^{2}={\left|S[l,m]\right|}^{2}{\left|H[l,m]\right|}^{2}+\left|A{\left[l,m\right]}^{2}\right|{\left|H[l,m]\right|}^{2}=\\ \left({\left|S[l,m]\right|}^{2}+\left|A{\left[l,m\right]}^{2}\right|\right){\left|H[l,m]\right|}^{2}\;,\; \end{array}$$

where, *S*[*l*,*m*] and *A*[*l*,*m*] are *s*(*n*) and *a*(*n*), respectively, in the Melspectrum domain. This shows that the *a*(*n*) component is additive on the *s*(*n*) component, whereas the stethoscope-induced multiplicative component nonlinearly affects both *a*(*n*) and *s*(*n*) in the Melspectrum domain.

Using the logarithm function on both sides of Equation (5), the Log-MelSpectrum features that can be expressed as *Log*-*Mels* variable in the formula:

(6) $$Log-Mels=\log{\left|Y[l,m]\right|}^{2}=\log{\left|H[l,m]\right|}^{2}+\log\left({\left|S[l,m]\right|}^{2}+\left|A{\left[l,m\right]}^{2}\right|\right).$$

Equation (6) shows that the stethoscope-induced multiplicative component can be converted into an additive term in the Log-MelSpectrum domain; that is, the Log-MelSpectrum feature after logarithmic transformation can represent the multiplicative noise as an additive component in the feature space. Meanwhile, from the study, we established that if the training data is overlaid with irrelevant additive noise and enough data are available for the model to converge, CNN is robust to additive noise. Therefore, Log-Melspectrum feature maps are easier to improve the classification performance of CNN and enhance the robustness of the model in different domains. This conclusion is further verified by the experiments described in the following section.

## 3. Experiments and Results

### 3.1. Heart-Sound Datasets

The heart-sound dataset used in our experiments was obtained from the 2016 PhysioNet/Computing in Cardiology (CinC) Challenge [33]. This dataset includes six sub-datasets: dataset-a, dataset-b, dataset-c, dataset-d, dataset-e, and dataset-f. Detailed information of these datasets is presented in Table 2. The distributions of these datasets are quite different. Specifically, dataset-e collected by MLT201/Piezo and 3 M Littmann made up approximately 66% of the total datasets, whereas dataset-c collected by AUDIOSCOPE accounted for only 1.7%. The distribution of the datasets varied with different acquisition equipment. Thus, the datasets had domain differences, making it difficult to classify heart sounds. 

### 3.2. CNN Architecture

**Figure 4:**
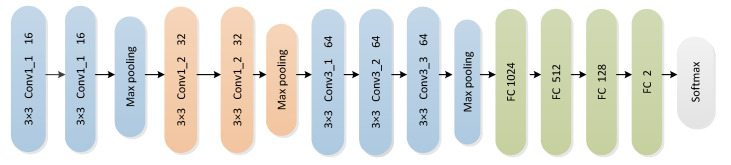
Modified VGG16 structure.

Regardless of the network parameters and training speed, we chose a general convolution network to classify the heart-sound fragments. VGG16, a simple but effective convolutional network, has been widely used in the field of face recognition and image classification [34]. Therefore, we used the VGG16 network to perform the task of heart-sound signals classification. VGG16 consists of a simple stack of seven 3 × 3 convolution layers (CLs), four fully-connected layers (FLs), and three maximum pooling layers (MLs). However, the input feature vector size was 128 × 128 and the original input feature vector size was 224 × 224. In addition, the VGG16 model structure was appropriately adjusted to process the heart-sound feature maps. The modified structure of VGG16 is shown in Figure 4. In this modified structure, the first maximum pooling layer is used to reduce the dimension of the previous input from 128 *×* 128 to 64 × 64, while the second maximum pooling layer is used to reduce the dimension of the previous input from 64 *×* 64 to 32 × 32. The third maximum pooling layer is used to reduce the dimension of the previous input from 32 *×* 32 to 16 × 16. Meanwhile, the last layer, which is a softmax layer, is connected to the normal and abnormal classes in the datasets. The convolution kernels move with the CNN training with the feature maps in the time and frequency axes, and the deep features are ultimately extracted from the heart sound signals in both frequency and time dimensions.

### 3.3. Experimental Process

**Figure 5:**
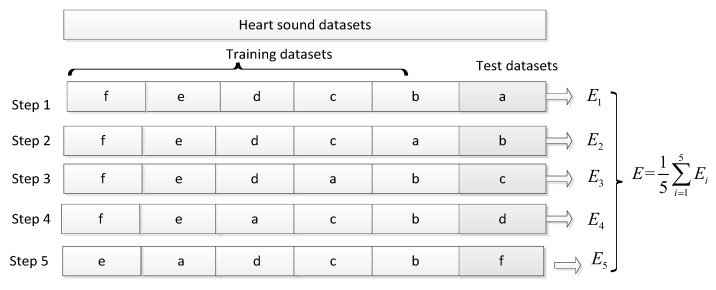
Experimental Process.

Five experiments were conducted to evaluate the stability and generalisation performance of the heart-sound classification method. Specifically, one dataset was selected from the data subset-a, -b, -c, -d, and -f in each experiment as test data, and the rest of the heart-sound subsets were used for training and optimisation of the model parameters. The data subset-e was only used for model training because it accounted for 66% of the total number of heart-sounds. The specific process is illustrated in Figure 5.

The CNN hyper-parameters yielded the best results, as presented in Table 3. In the training phase, 20% of the training datasets were used for model validation and an oversampling method was used to balance the normal and abnormal heart-sound samples. In addition, the Kaiming method was used to initialise the parameters and make the gradient of the learned parameters valid or saturated in the training phase.

### 3.4. Experimental Results and Analysis

#### 3.4.1. Model Training Results and Analysis

**Figure 6:**
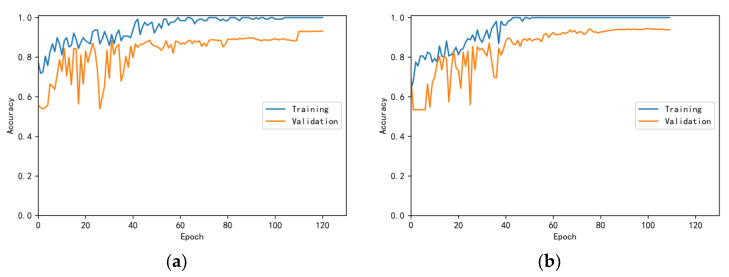
Training and validation accuracy curves on test dataset-a: (**a**) Log-MelSpectrum and (**b**) MelSpectrum feature maps as inputs.

**Figure 7:**
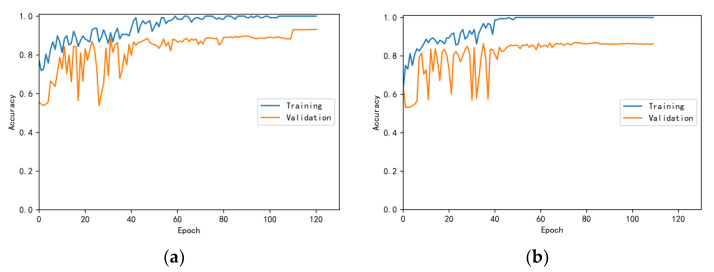
Training and validation accuracy curves on test dataset-b: (**a**) Log-MelSpectrum and (**b**) MelSpectrum feature maps as inputs.

**Figure 8:**
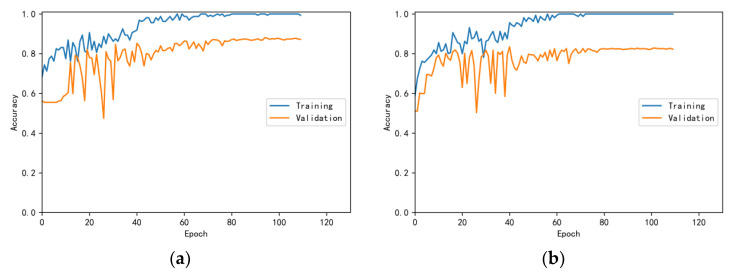
Training and validation accuracy curves on test dataset-c: (**a**) Log-MelSpectrum and (**b**) MelSpectrum feature maps as inputs.

**Figure 9:**
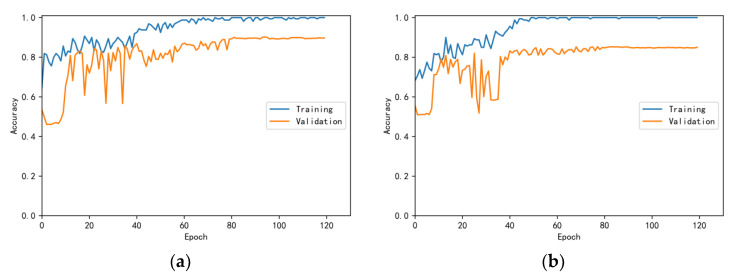
Training and validation accuracy curves on test dataset-d: (**a**) Log-MelSpectrum and (**b**) MelSpectrum feature maps as inputs.

**Figure 10:**
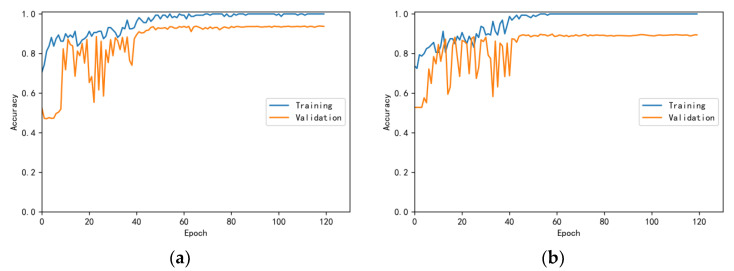
Training and validation accuracy curves on test dataset-f: (**a**) Log-MelSpectrum and (**b**) MelSpectrum feature maps as inputs.

**Figure 11:**
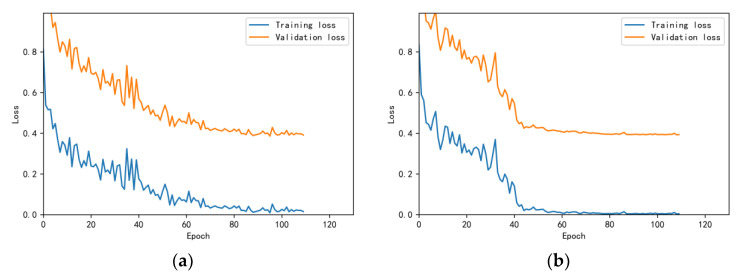
Loss curves on test dataset-a: (**a**) Log-MelSpectrum and (**b**) MelSpectrum feature maps as inputs.

**Figure 12:**
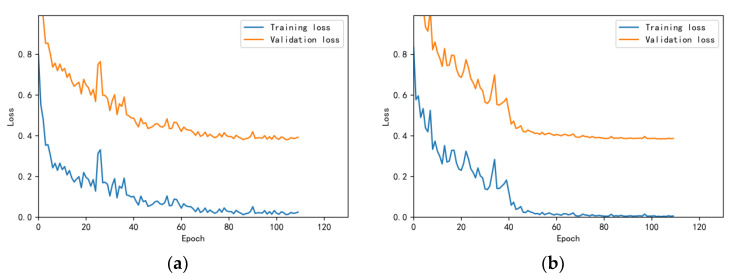
Loss curves on test dataset-b: (**a**) Log-MelSpectrum and (**b**) MelSpectrum feature maps as inputs.

**Figure 13:**
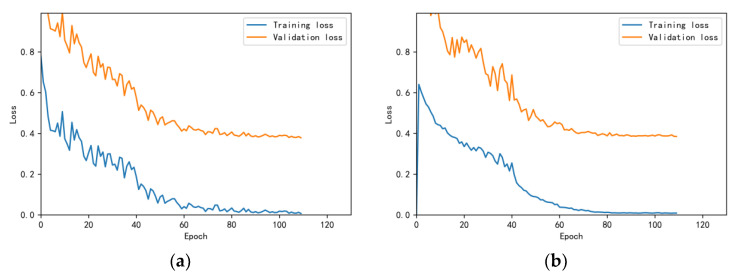
Loss curves on test dataset-c: (**a**) Log-MelSpectrum and (**b**) MelSpectrum feature maps as inputs.

**Figure 14:**
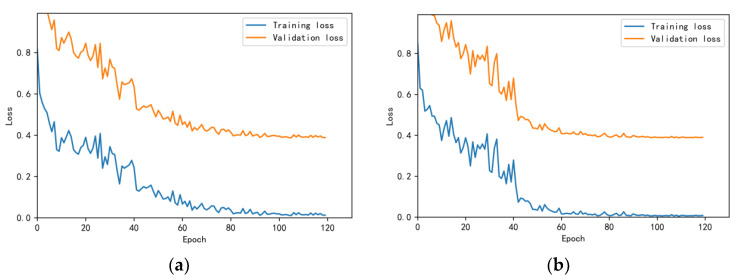
Loss curves on test dataset-d: (**a**) Log-MelSpectrum and (**b**) MelSpectrum feature maps as inputs.

**Figure 15:**
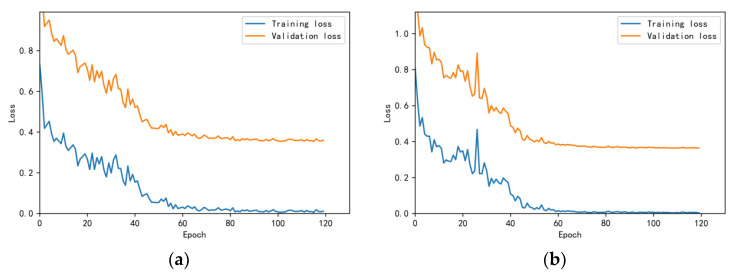
Loss curves on test dataset-f: (**a**) Log-MelSpectrum and (**b**) MelSpectrum feature maps as inputs.

The training and validation accuracy learning curves obtained by the CNN under different training datasets are shown in Figure 6, Figure 7, Figure 8, Figure 9 and Figure 10. The different curves show that as the number of iterations increases, the accuracy of training and validation gradually improve and become stable. The accuracies of the MelSpectrum feature maps on the validation dataset-a, dataset-b, dataset-c, dataset-d, and dataset-f were 97.0%, 86.4%, 82.3%, 85.0%, and 89.5%, respectively. The accuracies of the Log-MelSpectrum feature maps for the five validation datasets were 93.9%, 93.2%, 87.25%, 89.7%, and 93.7%, respectively. The loss curves for training and validation on the different datasets are shown in Figure 11, Figure 12, Figure 13, Figure 14 and Figure 15. As observed, the loss value of the model decreases with an increase in the number of iterations and eventually stabilises. The parameters of the model were set at realistic levels based on the accuracy and loss curves, and there was no overfitting or underfitting.

The validation accuracies are presented in Table 4. The accuracies of the Log-MelSpectrum and MelSpectrum time-frequency characteristic diagram are 91.74% ± 3.72% and 87.42% ± 3.99%, respectively. Therefore, the Log-MelSpectrum and MelSpectrum time-frequency feature maps discussed in this section can be used as feature input vectors for the CNN, which is an effective heart-sound classification method. Furthermore, Log-MelSpectrum features are more suitable for processing heart-sound datasets that have domain differences and for improving the performance of CNN for heart-sound classification, compared with MelSpectrum features. 

#### 3.4.2. Test Results and Analysis

**Figure 16:**
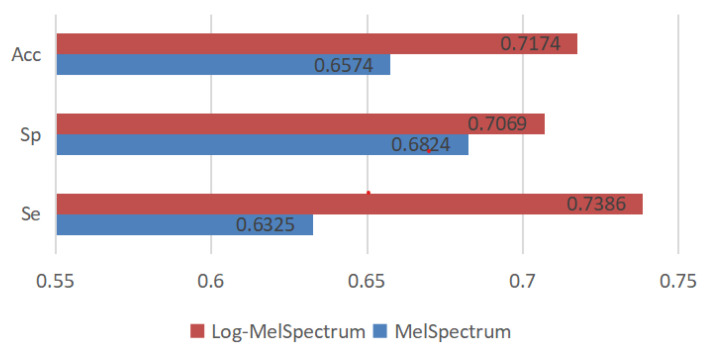
Average Performance of the Model.

The model performance results based on test dataset-a, dataset-b, dataset-c, dataset-d, and dataset-f are presented in Table 5. Specificity (Sp), Sensitivity (Se), and the mean of Se and Sp (MAcc) were used as evaluation indices in this study, as defined in [33]. Based on the results, deep learning models that were trained using different input time-frequency features gave different prediction results on the same test datasets. In our experiments, MAcc indices on test dataset-a, dataset-b, dataset-c, dataset-d, and dataset-f were 57.83%, 75.98%, 70.24%, 60.05%, and 64.61%, respectively, for MelSpectrum feature maps as the input, and 67.65%, 83.25%, 72.32%, 68.92%, and 66.54%, respectively, for Log-MelSpectrum feature maps as the input. Figure 16 shows the average performance of the model. The model trained by the Log-MelSpectrum feature maps has higher average Se, Sp, and MAcc than that trained by the MelSpectrum feature maps.

Based on the accuracy of the validation datasets and the test results for each test dataset, using either MelSpectrum or Log-MelSpectrum as input features of the CNN can be effective methods for classifying heart sounds. Furthermore, the Log-MelSpectrum feature maps can easily improve the classification performance of the model and enhance its robustness in different domains, compared with the MelSpectrum feature maps. This is because the Log-MelSpectrum feature maps can represent the multiplicative noise caused by the stethoscope as an additive component in the feature space, and the CNN is more robust to the noise of additive components.

## 4. Discussion

Heart sounds that can reflect the information of cardiovascular hemodynamic changes to diagnose CVDs. It is of great value to use a computer to extract the features from heart-sound signals for quantitative analysis. The most commonly used are MelSpectrum features and Log-MelSpectrums features. Systems based on CNNs trained on MelSpectrums and Log-MelSpectrums of segmental heart-sound signals are superior to other systems using hand-crafted features. However, no a priori evidence exists regarding the best input representation for classifying heart sounds when using CNN models. 

In this study, different input feature representations, including MelSpectrum and Log-MelSpectrum feature maps, are analysed to determine the most suitable method for classifying heart-sound signals when using CNNs. In particular, MelSpectrum and Log-MelSpectrum feature maps are discussed combined with the mathematical model of cardiac-sound acquisition. Based on theoretical analysis, heart-sound signals are always disturbed by additive and multiplicative noises. The multiplicative noises are due to the stethoscopes, and stethoscope-induced multiplicative noises can be converted into an additive term in Log-MelSpectrum domain. Hence, Log-MelSpectrum feature maps can transform nonlinear additive noise into linear noise. Moreover, the CNN is robust to the additive noise of the input layer. Therefore, we conclude that the Log-MelSpectrum feature maps as the input feature vector of the CNN can efficiently suppress the additive noise. This conclusion is further validated in our experiments.

In the five different experiments, MelSpectrum and Log-MelSpectrum feature maps were input to train a modified CNN. The accuracies of the Log-MelSpectrum feature maps on the validation dataset-a, dataset-b, dataset-c, dataset-d, and dataset-f were all higher than those using the MelSpectrum feature maps in the experiments and the variance of mean accuracies using the Log-MelSpectrum as inputs were less than those using the MelSpectrum as inputs. Furthermore, the model trained by the Log-MelSpectrum feature maps has higher accuracy in terms of average Se, Sp, and MAcc than that trained by the MelSpectrum feature maps. The experimental results showed that using the feature maps of MelSpectrum and Log-MelSpectrum as inputs to the CNN can be effective methods for classifying heart sounds. Furthermore, Log-MelSpectrum features are more suitable for processing heart-sound datasets that have domain differences and for improving the performance of CNN for heart-sound classification compared with MelSpectrum features.

The average sensitivity and specificity on testing datasets trained by the Log-MelSpectrum feature maps are 73.86% and 70.69%, respectively. The result is lower than that of Maknickas [19] and Li [32]. This may be due to the following reasons. First, the mode proposed by Maknickas is deeper than ours and deep learning models with deeper layers normally exhibit more accurate performance, and this has been the tendency in recent developments. Second, Li improved Log-MelSpectrum feature maps using dynamic and static MelSpectrum features, and used them as input features for deep residual learning. Although the sensitivity and specificity levels on testing datasets are far away from a useful diagnostic model in clinical settings, our work mainly resolved the issue that STFT-based features are more suitable for classifying normal and abnormal heart sound signals. As far as we know, this is the first study that analysed theoretically the MelSpectrum and Log-MelSpectrum features of heart-sound signals to determine which one is more suitable for classifying heart-sound signals when using CNNs. We believe the study provided a solid solution in the field of heart-sound classification and could promote the automatic diagnosis of CVDs.

## 5. Conclusions and Future Work

The intelligent classification of heart-sound signals can assist clinicians in the rapid diagnosis of CVDs. In this study, the STFT-based features including MelSpectrum and Log-MelSpectrum were investigated and confirmed as being suitable for classifying normal and abnormal heart sound signals when using CNNs. Both the experimental results and theoretical analysis demonstrate that the Log-MelSpectrum can reduce the classification difference between domains and improve the performance of CNN for heart-sound classification.

Although the study provided a significant contribution to the field of heart-sound classification in promoting the diagnosis of CDVs, there are still limitations that necessitate further work. First, other techniques such as continuous wave transformations and linear prediction cepstrum coefficients can be analysed and compared with STFT-based features in the application of heart-sound classification. Second, a standardized heart sounds database for specific heart diseases should be established to provide training data for artificial intelligence algorithms with strong robustness and high accuracy in practical application scenarios.

## Figures and Tables

**Table 1:** Detailed parameters of MelSpectrum and Log-MelSpectrum features.

Parameter	Value	Description
Low and high frequency	25 Hz to 950 Hz	The band frequency of Butterworth filter
Window_function	Hanning	Window function selected in FFT operation
Hop_length	60	Number of samples between consecutive frames
Sampling_frequency	2000 Hz	Sampling frequency
N-FFT	512	The length of FFT operation
Window_size	240	Frame length in FFT operation
Sample_size	2.5 s	The length of heart sound signals selected from the start position the cardiac cycle
Mel_filters	128	The number of Mel filters

**Table 2:** Heart-sound datasets used in our experiments.

Subset	Normal Recordings	Abnormal Recordings	Account for Total Datasets	Acquisition Equipment
a	117	292	12.62%	Welch Allyn Meditron
b	386	104	15.12%	3 M Littmann E4000
c	7	24	0.96%	AUDIOSCOPE
d	27	28	1.70%	Infral Corp. Prototype
e	1958	183	66.08%	MLT201/Piezo, 3 M Littmann
f	80	34	3.52%	JABES
Total	2575	665	100%	

**Table 3:** Initial hyper-parameters of the CNN.

Parameter	Step 1	Step 2	Step 3	Step 4	Step 5
Training datasets/Test datasets	b, c, d, e, f/a	a, c, d, e, f/b	a, b, d, e, f/c	a, b, c, e, f/d	a, b, c, d, e/f
Learning rate	0.0001	0.0001	0.0001	0.0001	0.0001
Epoch_size	25	22	26	26	25
Epoch	110	110	110	120	120
BatchSize	160	160	160	160	160
Optimiser	Adam	Adam	Adam	Adam	Adam
Loss function	Cross-Entropy	Cross-Entropy	Cross-Entropy	Cross-Entropy	Cross-Entropy

**Table 4:** Validation accuracy of the model under different test datasets.

Input	Dataset- a	Dataset- b	Dataset- c	Dataset- d	Dataset- f	Meana Ccuracy+ Variance
Log- MelSpectrum	**97.0%**	**93.0%**	**87.3%**	**87.7%**	**93.7%**	**91.74 + 3.72**
MelSpectrum	93.9%	86.4%	82.3%	85%	89.5%	87.42 + 3.99

**Table 5:** Performance of the model based on different test datasets.

Input Feature	Se	Sp	MAcc
Performance of the model based on test dataset-a			
MelSpectrum	0.6267	0.5299	0.5783
Log-MelSpectrum	0.5582	0.7949	**0.6765**
Performance of the model based on test dataset-b			
MelSpectrum	0.5714	0.9482	0.7598
Log-MelSpectrum	0.7143	0.9508	**0.8325**
Performance of the model based on test dataset-c			
MelSpectrum	0.8333	0.5714	0.7024
Log-MelSpectrum	0.875	0.5714	**0.7232**
Performance of the model based on test dataset-d			
MelSpectrum	0.5714	0.6293	0.6005
Log-MelSpectrum	0.7857	0.5926	**0.6892**
Performance of the model based on test dataset-f			
MelSpectrum	0.5588	0.7333	0.6461
Log-MelSpectrum	0.7059	0.625	**0.6654**

## Data Availability

The original heat-sound datasets are available from https://physionet.org/content/challenge-2016/1.0.0/#files (accessed on 1 July 2021).
